# Population dynamics of little brown bats (*Myotis lucifugus*) at summer roosts: Apparent survival, fidelity, abundance, and the influence of winter conditions

**DOI:** 10.1002/ece3.7573

**Published:** 2021-05-07

**Authors:** Robert A. Schorr, Jeremy L. Siemers

**Affiliations:** ^1^ Colorado Natural Heritage Program Colorado State University Fort Collins CO USA

**Keywords:** abundance, fidelity, little brown bat, mark–recapture, maternity roost, *Myotis lucifugus*, population dynamics, summer roost, survival

## Abstract

1. White‐nose syndrome (WNS) has caused the death of millions of bats, but the impacts have been more difficult to identify in western North America. Understanding how WNS, or other threats, impacts western bats may require monitoring other roosts, such as maternity roosts and night roosts, where bats aggregate in large numbers.
2. Little brown bats (*Myotis lucifugus*) are experiencing some of the greatest declines from WNS. Estimating survival and understanding population dynamics can provide valuable data for assessing population declines and informing conservation efforts.
3. We conducted a 5‐year mark–recapture study of two *M. lucifugus* roosts in Colorado. We used the robust design model to estimate apparent survival, fidelity, and abundance to understand population dynamics, and environmental covariates to understand how summer and winter weather conditions impact adult female survival. We compared the fidelity and capture probability of *M. lucifugus* between colonies to understand how bats use such roosts.
4. Overwinter survival increased with the number of days with temperatures below freezing (*β* > 0.100, *SE* = 0.003) and decreased with the number of days with snow cover (*β* < −0.40, *SE* < 0.13). Adult female fidelity was higher at one maternity roost than the other. Overwinter and oversummer adult female survival was high (>0.90), and based on survival estimates and fungal‐swabbing results, we believe these populations have yet to experience WNS.
5. Recapture of *M*. *lucifugus* using antennas that continuously read passive integrated transponder tags allows rigorous estimation of bat population parameters that can elucidate trends in abundance and changes in survival. Monitoring populations at summer roosts can provide unique population ecology data that monitoring hibernacula alone may not. Because few adult males are captured at maternity colonies, and juvenile males have low fidelity, additional effort should focus on understanding male *M. lucifugus* population dynamics.

## Graphical abstract

This is a mark–recapture analysis of little brown bat populations at two summer roosts to understand abundance, survival, and fidelity. At the maternity colony, adult females showed high survival and fidelity, while another colony that may serve as a maternity, day, and night roosts had lower estimates of fidelity.

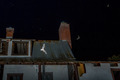

## 1 INTRODUCTION

Informed management and conservation of wildlife populations necessitate understanding the status and trend in population parameters, and the uncertainty of those parameters. For many nongame species, there is limited information regarding survival, abundance, and recruitment that would drive decision making for management or conservation strategies (Thompson, 2004). Knowing baseline population parameters, and the variability of such parameter estimates, allows comparisons as resource availability fluctuates or as new threats arise (Runge, 2011). When species are experiencing precipitous declines, the population changes can be obvious, yet without understanding of prior population conditions, it is challenging to devise appropriate population recovery goals (Mills, 2007). The population ecology of some wildlife species, such as bats, is particularly hard to study (O'Shea et al., 2003), and even when species are prioritized for conservation, the limited or biased understanding of population dynamics can hinder conservation action (Weller et al., 2009).

For 15 years, North American bat populations have declined at unprecedented rates because of white‐nose syndrome (WNS; Blehert et al., 2009; Frick, Pollock, et al., 2010). First observed in northeastern United States in 2007, WNS is an introduced fungal disease that has spread throughout much of eastern and central North America, causing the mortality of millions of bats (Hoyt et al., 2021). As WNS has spread westward, biologists have struggled to estimate the severity of bat population declines. Because overwinter roosts (hibernacula) with large populations are scarce in western North America, biologists have to rely on sampling during the summer when bats are more accessible and aggregate in large numbers (Weller et al., 2018). Biologists are passively recording bats' ultrasonic vocalizations while they forage, or sampling summer roosts where bats are more easily observed (Loeb et al., 2015; Weller et al., 2009). For example, acoustic recordings allow landscape‐scale occupancy monitoring to understand changes in broad‐scale distribution, diversity, occupation dynamics, and regional population change (Rodhouse et al., 2019). However, these landscape‐scale approaches are not sensitive to detecting local population declines (Conner et al., 2016). Estimates of local population demographic parameters are necessary for evaluating the underlying changes that affect bat population dynamics. Individual‐based mark–recapture approaches can assess local population status prior to WNS exposure and identify population‐level impacts after threats arrive (Frick et al., 2010; Loeb et al., 2015; Maslo et al., 2015).

To understand bat population changes in North America, a continental bat monitoring program (NABat) employs a broad‐scale resolution to assess landscape‐scale patterns and a finer‐scale resolution to understand site‐specific population dynamics (Reichert et al., 2021). Much of the broad‐scale monitoring incorporates nationwide summer acoustic recordings, while the fine‐scale monitoring uses colony counts at known roosts, such as hibernacula and maternity colonies (Loeb et al., 2015). For species that are poorly detected by acoustic sampling (Kaiser & O'Keefe, 2015), or where hibernacula are difficult to find (Weller et al., 2018), summer‐colony counts can be an effective monitoring strategy. Maternity colonies, where large numbers of individuals aggregate, can reliably provide sample sizes for assessing long‐term trends, but monitoring at these roosts may neglect males (Weller et al., 2009). Monitoring a suite of summer roosts, such as maternity roosts, day roosts, and night roosts, may elucidate trends among all ages and sexes, and identify conservation strategies for different demographics of the population. Additionally, understanding how different demographic groups use nonmaternal summer roosts can clarify demographic‐specific energy budgets, roosting needs, and local resource needs (Anthony et al., 1981; Weller et al., 2009).

The breadth and scale of impacts from WNS may be heightened for North American bats as climate change produces less‐predictable seasonal climatic patterns, with greater seasonal physiological stress (Williams et al., 2015). To survive winter, when insect prey are unavailable, hibernating bats dramatically lower their body temperatures and slow their metabolism to reduce energy consumption (Geiser, 2004). Bats will accumulate fat prior to hibernating and then select winter roosts with stable, cold temperatures that minimize energetic losses (Boyles et al., 2007; Perry, 2013). External environmental factors can influence the availability of such roosts, and thus, the rate bats use energetic reserves (Humphries et al., 2002). Accumulating and retaining fat are necessary for hibernating bats' overwinter survival, but an additional benefit is the reduction in mortality rate for WNS‐infected individuals (Cheng et al., 2019). If late‐winter and early‐spring weather conditions become erratic with periods of warming and cooling, bats may emerge from hibernation prior to food availability, or be unable to emerge and feed because of late snowfall (Rodenhouse et al., 2009). Abnormal climate patterns may limit bats' ability to reduce energetic losses, retain fat reserves, and fight off WNS infections (Hayman et al., 2016; Jonasson & Willis, 2011). Elucidating the impact environmental conditions have on bat survival can help biologists understand how climatic variability may jeopardize bat conservation efforts.

**FIGURE 1:**
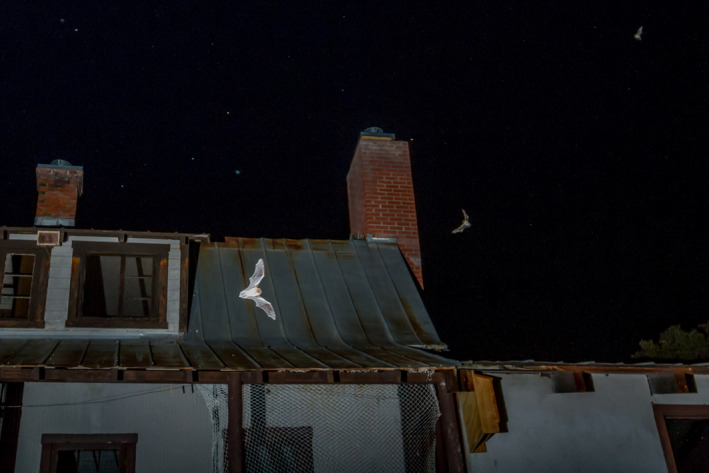
Little brown bats (*Myotis lucifugus*) in front of the Rehder Ranch house maternity colony. Photograph by George Fargo

Historically, the little brown bat (*Myotis lucifugus*; Figure 1) was assumed to be the most broadly distributed, and arguably the most abundant, North American bat species throughout much of the United States and Canada (Fenton & Barclay, 1980; Kunz & Reichard, 2010). Unfortunately, since WNS arrival in North America, eastern *M. lucifugus* populations have declined by millions, with population projections suggesting regional extinction in northeastern United States (Frick, Pollock, et al., 2010; Kunz & Reichard, 2010). Now that WNS has arrived in western states (Lorch et al., 2016), wildlife biologists are anxious to understand how the disease is impacting *M. lucifugus* populations. Thus, landscape, regional, and local population monitoring programs are being instituted to understand distribution and abundance of *M. lucifugus* prior to, and after the arrival of, WNS (Frick, Reynolds, et al., 2010; Loeb et al., 2015; Rodhouse et al., 2019). Because WNS impacts bats overwinter, there is a priority to understand populations in hibernacula, but given the scarcity of these roosts in western North America, attention has focused on monitoring summer colonies (Weller et al., 2018).

To better understand abundance and survival of *M. lucifugus* prior to WNS, we compare fidelity and population dynamics of *M. lucifugus* at different summer roost types, and understand the impacts seasonal weather has on *M. lucifugus* survival, we conducted a mark–recapture study at two colonies. We use 5 years of mark–recapture data at a maternity colony and a night roost to (a) estimate vital population parameters, such as survival and abundance; (b) assess the impact of environmental factors on seasonal survival of *M. lucifugus*; and (c) estimate the fidelity of individuals to particular roosts.

## 2 MATERIALS AND METHODS

**FIGURE 2:**
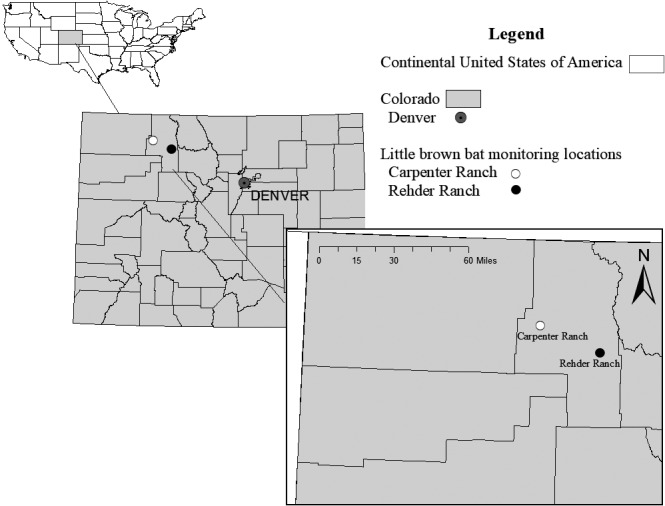
Location of little brown bat (*Myotis lucifugus*) barn roost (Carpenter Ranch) and house roost (Rehder Ranch) in northwestern Colorado, USA

We conducted this study at two roosts within the Yampa Valley of northwest Colorado (Figure 2). One colony (herein called “house roost”) is in a 120‐m^2^ ranch house at the Rehder Ranch (elevation 2,150 m) built in 1900. The house roost is adjacent to Catamount Lake and approximately 17 km south of Steamboat Springs, Colorado. Bats roost in the spaces between the interior walls and exterior metal roofing material. The other colony (herein called “barn roost,” elevation 1,930 m) is in the Carpenter Ranch barn, which was built in 1903, is adjacent to the Yampa River, and is approximately 7 km east of Hayden, Colorado. Bats roost between timbers in the hayloft and between timbers and the metal roof.

We captured bats using harp traps and mist nets around the outside of the house roost and within the barn roost. We captured bats in early summer (June) and when young of the year (juveniles) were believed to be volant, but had not dispersed (late summer: late July/early August). We captured and marked bats twice a year from 2014 to 2018, except for late summer of 2017 at the house roost. Because the timing of *M. lucifugus* parturition varies each year, the timing of the late‐summer capture event was adjusted to optimize juvenile captures. For each bat captured, we recorded the mass, sex, and age as juvenile or adult (Brunet‐Rossinni & Wilkinson, 2009), palpated and assessed the individual's reproductive condition, and marked it with HPT9 (9 mm × 2 mm) 134.2 kHz passive integrated transponder (PIT) tag (Biomark, Inc., Boise, Idaho). We inserted tags subcutaneously below the scapula, and we sealed insertion sites with a biomedical glue (Vetbond Tissue Adhesive; 3 M Science, St. Paul, Minnesota). We conducted facial and wing swabbing and guano collection to assess the presence of the fungus (*Pseudogymnoascus destructans*; *Pd*) that causes WNS (analysis conducted by U.S. Geological Survey, National Wildlife Health Center, Madison, WI, USA).

At the house roost, we detected tagged bats using a 7.6‐m cord PIT‐tag antenna system (IS1001; Biomark, Inc., Boise, ID, USA; herein called “antenna”) that was stretched under the eaves. We installed this antenna in the summer of 2015, and it was disrupted for several weeks late in summer of 2015 and early 2016 because the solar panel became disconnected from the battery. At the barn roost, we varied the PIT‐tag reading system's configuration and placement over time. In 2014, we placed two 0.6 m × 0.6 m window‐style PIT‐tag readers (FS2001; Biomark, Inc.) at the top of the primary entrance (3.5 m × 3.5 m barn door opening), while netting was stretched along the lower section of the entrance to channel bat flight through or near the window‐style readers. This arrangement produced few detections (<60 detections/2 weeks), so in 2015, we installed a cord antenna system like that used at the house roost. We weaved the cord antenna across the barn door opening with approximately 0.25‐m gap, and then in August 2015 moved the antenna within the hayloft to reduce conflicts with ongoing cattle operations. At both locations, we acquired additional detections using handheld PIT‐tag readers to scan roosting bats in the hayloft of the barn roost, and at bat houses near the house roost.

We created encounter histories based on weeks of the year when individuals were captured or detected; thus, there were 52 encounter periods throughout the year. We structured the data into weekly intervals because it allowed multiple reencounter (recapture) occasions throughout the summer. These multiple opportunities to reencounter tagged animals increase the opportunities to detect bats and improve estimates of detectability. We analyzed mark–recapture data using a Huggins robust design model in Program MARK (Kendall, 2001). The robust design model allows estimation of parameters, such as abundance, during the closed sessions when we assume there are no births, deaths, immigration, or emigration. Also, the robust design model allows estimation of other parameters, such as survival, immigration, emigration, and fidelity, during open sessions. Because the fate of juvenile bats may be strongly associated with the fates of their mothers, we estimated an overdispersion scaling parameter (*ĉ*), from the global model in a Cormack–Jolly–Seber analysis (White & Burnham, 2001). We compared competing models using quasi‐likelihood Akaike's information criterion with small sample size bias correction (QAIC*_c_*) and the probability of a model being the most parsimonious model (QAIC*_c_* weight; *w_i_*—Burnham & Anderson, 2002).

We estimated oversummer and overwinter survival, because if WNS was impacting *M. lucifugus* populations, we expected overwinter survival to be lower than oversummer survival. To estimate oversummer survival, we structured the encounter data such that there was an open season between summer tagging events. To estimate overwinter survival, we used the open session between late summer and early summer tagging events. The two periods of population closure each year were based on the arrival of a substantial number of individuals (>30 individuals/week) after hibernation and the departure of most bats before hibernation (<30 individuals/week remaining at the roost). The selection of 30 individuals is arbitrary, but chosen to designate when we believed bats were returning to the roost. The “early‐summer closed period” began after the week when greater than 30 individuals were detected (late April to mid‐May) and extended until the week after the capture and tagging event in early June. The time between early‐summer (June) and late‐summer (late July/early August) capture events was considered open because young were being born. The “late‐summer closed session” began after the July/August capture event and ended the week when fewer than 30 individuals were detected in early fall (mid‐August to mid‐September).

Using the robust design model, we estimated capture probability (*p*), recapture probability (*c*), overwinter and oversummer apparent survival (*φ*), abundance (N), temporary immigration (1 − γ′), temporary emigration (γ″), and site fidelity (1 − γ″; the probability of being detected at time interval *i*, given you were detected in time interval *i* − 1). As a modeling approach, we first modeled capture and recapture probability, keeping apparent survival and movement parameters (γ′, γ″) as varying temporally. Once the best set of models of *p* and *c* was identified, we used those *p* and *c* configurations to model *φ*, γ′, and γ″. Seasonal parameter estimates were transformed based on weekly estimates, and variances were estimated using the delta method (Powell, 2007). Abundance at each roost was estimated for each of the two closed periods per year. For estimating juvenile *φ,* we transitioned juveniles into adults the first year after they were captured, when they would be sexually mature (Humphrey & Cope, 1976). We ran 27 models for *p* and *c*, and we ran 89 models for *φ* and the movement parameters using the most parsimonious model of *p* and *c*.

Because weather can impact bat activity and survival, we used weather data from a Steamboat Springs weather station (National Oceanic and Atmospheric Administration National Centers for Environmental Information, Station No. GHCND:USCOOO57936; ncdc.noaa.gov) to model capture and recapture probabilities, and monthly weather summaries to model seasonal survival. We used number of days with precipitation >0.25 and >2.5 cm, number of days with maximum temperature >21 and >32°C, and total monthly precipitation (in) to model oversummer *φ*. We used measures of winter severity, including number of days with low temperature <0°C, number of days with low temperature <−32°C, number of days with high temperature <0°C, number of days with precipitation >0.25 cm, number of days with precipitation >2.5 cm, number of days with snow depth ≥2.5 cm, and total precipitation as covariates for modeling overwinter survival. Additionally, we used the variance of winter covariates to assess how the variability of winter severity impacted parameters.

We used individual covariates of mass and body condition index (BCI: forearm length/mass) as covariates for seasonal survival (Pearce et al., 2008). Mass was measured at each capture event, but we did not begin measuring forearm lengths until 2016.

## 3 RESULTS

We manually captured 1,741 individuals (via harp traps and mist nets) and manually recaptured 180 individuals 240 times. Between tagging events and recaptures (via antennas), we had an effective sample size of 8,539 captures and recaptures. Three individuals were removed from analysis after we found their shed tags in the barn roost. A vast majority of manually captured individuals were adult females (78%), with some juveniles (20%), and few captures of adult males (2%) (Table 1). Most juveniles were manually captured only during the late‐summer sampling period (Table 1). The earliest date in a year that a tagged bat was detected by antennas was on 15 April 2016, and the latest was on 19 September 2017. A majority (1,440) of bats were encountered at antennas, but 17% (301) were not detected by antennas. Bat mass averaged 7.2 g (*SD* = 1.1 g, *n* = 1,696), forearm length averaged 39.2 mm (*SD* = 2.4, *n* = 847), and BCI averaged 0.176 (*SD* = 0.221, *n* = 847).

**TABLE 1:** Age and sex of little brown bats (*Myotis lucifugus*) captured at Carpenter Ranch barn roost and Rehder Ranch house roost, Colorado, 2014–2018. Sex and age for two bats were undetermined because they escaped after tagging

	2014		2015		2016		2017		2018		Total
	June	August	June	August	June	August	June	July	June	July	
Carpenter Ranch barn											
Female											
Adult	124	89	162	55	30	45	73	20	41	40	679
Juvenile	0	43	1	4	0	33	0	35	0	66	182
Male											
Adult	4	10	2	7	2	2	2	3	2	5	39
Juvenile	0	20	1	4	0	32	0	28	1	39	125
Undetermined				1		1					2
										Barn Total	1,027
Rehder Ranch house											
Female											
Adult	168	80	170	62	87	29	58	‐	84	120	858
Juvenile	0	16	0	3	0	12	0	‐	0	9	40
Male											
Adult	0	1	1	3	4	0	2	‐	1	2	14
Juvenile	0	17	0	5	0	8	0	‐	0	12	42
										House Total	954
										Total	1,981

The best model of *p* and *c* used covariates of type of roost (barn versus house), season (early summer versus late summer), and time (week). The top model (100% of *w_i_*) of *φ* and movement parameters treated female adults separately from other age/sex groups. Female adult *φ* was best modeled using covariates of the prior season's number of days with the low temperature at or below freezing (herein called “freezedays”), the number of days with snow cover >2.5 cm (herein called “snowdays”), and the interaction of these two factors. Survival of adult males and juveniles was modeled as constant over time. Female adult γ″ was best modeled as dependent on time, and adult males and juveniles were modeled as constant over time, while γ′ was best modeled by age/sex group, location, season, and time.

**FIGURE 3:**
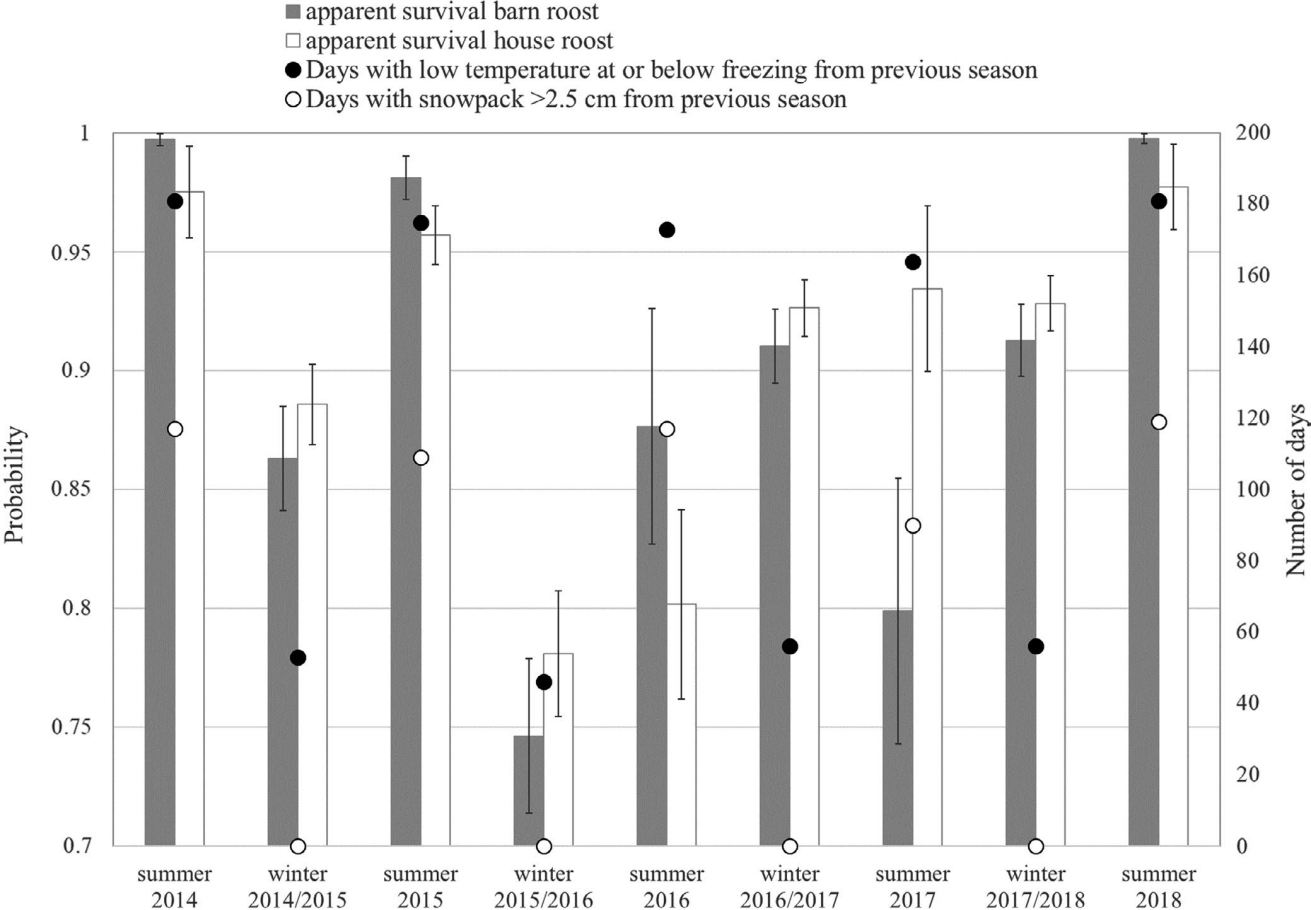
Adult female little brown bat (*Myotis lucifugus*) seasonal apparent survival (±*SE*), number of days with daily low temperature at or below 0°C, and number of days with snowpack >2.5 cm at the barn and the house roost, Colorado, USA, 2014–2018

Oversummer and overwinter adult female *φ* ranged from 0.75 to 0.99 (Figure 3) and was positively impacted by number of freezedays, negatively impacted by snowdays, and positively impacted by the interaction of freezedays and snowdays (Table 2). Estimates of juvenile female annual *φ* from the barn roost and the house roost were 0.45 (±0.06) and 0.71 (±0.09), respectively. Adult male yearly *φ* at the barn was 0.62 (±0.10 *SE*), but there were too few adult males captured at the house roost to produce reliable variance estimates. Estimates of juvenile male annual *φ* from the barn and house roosts were 0.53 (±0.29) and 0.07 (±0.51), respectively, and, based on the large variance estimates, indicate that too few juvenile males returned as adults to produce reliable estimates.

**TABLE 2:** Impact (logit β) of environmental covariates on adult female little brown bat (*Myotis lucifugus*) survival, Colorado, 2014–2018

Location	Environmental covariate	Estimate	SE	LCI	UCI
Carpenter Ranch barn	Number of days below freezing	0.106	0.003	0.099	0.112
	Number of days with snowfall >1 in	−0.716	0.101	−0.913	−0.519
	interaction of freezing days and snow days	0.003	0.001	0.002	0.005
Rehder Ranch house	Number of days below freezing	0.109	0.003	0.104	0.115
	Number of days with snowfall >1 in	−0.406	0.128	−0.657	−0.155
	interaction of freezing days and snow days	0.002	0.001	0.000	0.003

Abbreviations: LCI, lower 95% confidence interval; SE, standard error; UCI, upper 95% confidence interval.

**FIGURE 4:**
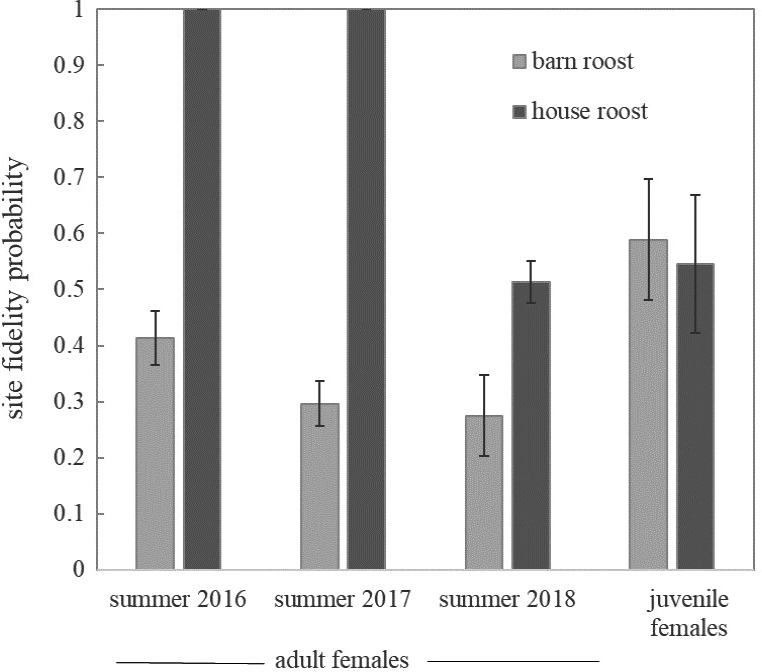
Probability (±*SE*) of summer site fidelity of adult female and annual site fidelity of juvenile female little brown bats (*Myotis lucifugus*) at the barn and the house roosts, Colorado, USA, 2016–2018

**FIGURE 5:**
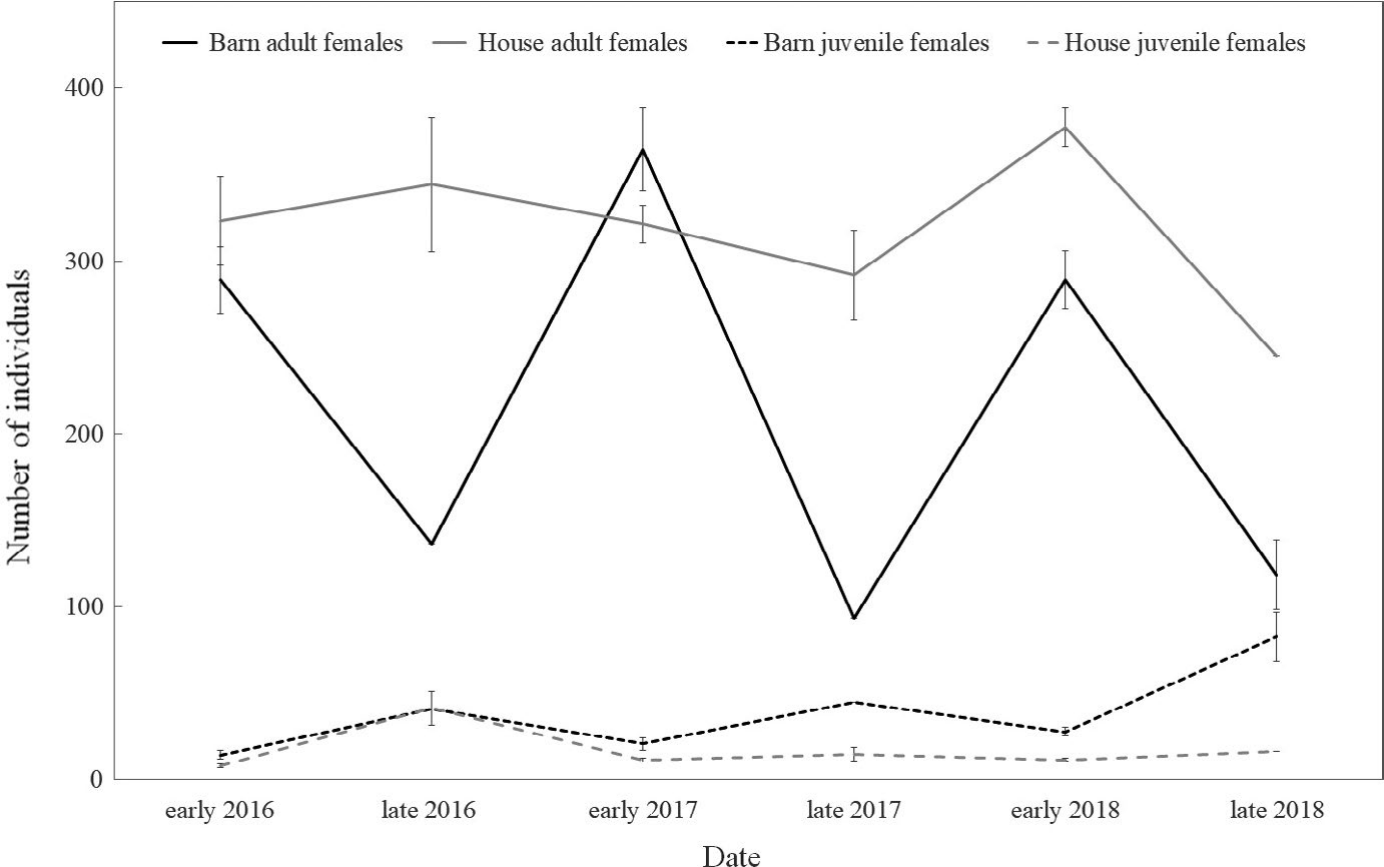
Summer abundance (±*SE*) of adult and juvenile female little brown bats (*Myotis lucifugus*) at the barn and the house roosts, Colorado, USA, 2016–2018

Estimates of site fidelity and abundance prior to 2016 were poorly estimated and highly variable. After 2015, when window‐frame antennas were no longer used, power loss issues were resolved, cord antennas were positioned to increase detections, and abundance and site fidelity were better estimated. The probability of site fidelity was high for adult females in summer (range: 0.48–0.90), with higher fidelity rates at the house roost (Figure 4). Site fidelity for juvenile females ranged from 0.55 to 0.59 (Figure 4). For males, fidelity was not estimable or had incredible estimates of variance (0 or 1). Abundance estimates of adult females ranged between 100 and 400 at the barn roost and 250 and 375 at the house roost (Figure 5). Estimates of juvenile female abundance were consistently low throughout the study. Too few males were detected to estimate abundance accurately.

During late spring and early summer, *p* at both roosts was stable and low (0.05–0.25), until after the first tagging even in early June when there was an influx of newly tagged individuals. During this same time, *c* stays high (0.50–0.78) at both roosts. During the latter half of the summer, *p* at the house roost is high (max = 0.99), but *c* steadily decreases until August when both *p* and *c* are <0.05. At the barn roost, *p* and *c* slowly decrease after the late‐summer tagging event and *p* became highly variable.

Models that included individual covariates were uninformative. Because mass and BCI can only be collected while the bat is in‐hand, all of the recaptures from antennas are missing individual covariate data.

## 4 DISCUSSION

Adult female annual survival from these Colorado roosts (range: 0.73–0.87) was comparable to *M. lucifugus* populations prior to exposure to WNS and higher than a population recovering from WNS (Frick, Pollock, et al., 2010; Maslo et al., 2015). A 16‐year study of a pre‐WNS exposure *M. lucifugus* colony in New Hampshire found adult female annual survival ranged from 0.63 to 0.90 (Frick, Reynolds, et al., 2010). A *M. lucifugus* roost in New Jersey recovering from WNS had lower adult female survival (range: 0.65–0.70; Maslo et al., 2015). Based on the estimates of survival at these Colorado roosts, and the absence of evidence of *Pd*, we believe WNS has not impacted these colonies. However, these roosts are only 270 km from a *Pd*‐infected colony in southeastern Wyoming (O'Driscoll et al., 2018), and WNS is expected to spread to Colorado eventually (Maher et al., 2012). These estimates provide a baseline understanding of *M. lucifugus* survival and can aid in assessing the impacts should WNS arrive. Should annual survival rates start to resemble those estimated at WNS‐infected colonies (Maslo et al., 2015), conservation measures that minimize population decline can be initiated.

Oversummer adult female apparent survival is impacted by the weather patterns in the previous year. The number of cold days (freezedays) from the previous winter was correlated with adult female *M. lucifugus* summer survival. Females that experience stable overwinter conditions may arrive in better physiological condition, better prepared for early parturition, and able to take advantage of sporadic insect availability of cool spring days (Norquay & Willis, 2014; Rydell, 1989). Additionally, stability of cold overwinter temperatures and humidity can provide the microclimates for energy conservation and increased likelihood of survival (Boyles et al., 2007; Jonasson & Willis, 2011; Kunz et al., 1998). Although we expected snowpack consistency to provide reliable thermal insulation for hibernating bats, the number of days with snowpack (snowdays) negatively impacted survival, and it is possible that late fall or early‐spring snowfall can prolong hibernation, exhaust fat reserves, prevent early emergence from hibernation, and preclude feeding bouts that build or replenish hibernation energy stores (Norquay & Willis, 2014; Paige, 1995). Increased snowpack days may inhibit bats' ability to periodically emerge from hibernacula because consistent snow cover traps them in high‐elevation retreats (Neubaum, 2018). Historically, Colorado's high‐elevation mountains have shown consistent snowpack and temperatures, but recently increased spring warming increases snowmelt, even while spring typically delivers periodic snowfall (Clow, 2010; Harrington et al., 1987). The increasing variability of weather in spring has the compounding effect of regularly dropping barometric pressure that can awaken torpid bats (Paige, 1995), while extending winter season length that jeopardizes survival (Besler & Broders, 2019; Czenze & Willis, 2015). This environmental variability can be energetically expensive and reduce bat survival (Thomas et al., 1990).

The positive influence of the interaction of freezedays and snowdays suggests the availability of blanket of insulating snow, and the cold days that increase its permanence, may be valuable for providing consistency in the thermal or humidity environments for hibernating bats (Boyles et al., 2007). In eastern North American caves, hibernating female *M*. *lucifugus* have shown resiliency to warming roost temperatures (Norquay & Willis, 2014); however, if *M. lucifugus* in western North America are using crevice or scree roosts as hibernacula (Neubaum et al., 2006; Weller et al., 2018), their insulation from changing environmental conditions may be less substantial than for cave‐ or mine‐roosting bats. Using weather stations in proximity to summer colonies is only a proxy for the roosting conditions bats may be experiencing, as there is growing evidence *M. lucifugus* fly considerable distances to overwinter at higher elevations (Neubaum, 2018; Norquay et al., 2013). Using hibernacula‐specific weather covariates of temperature and snowpack may clarify the role winter conditions play in seasonal survival.

Although Colorado, and many western states, has an abundance of mines and caves where bats reside, there are few known roosts with large numbers of overwintering bats (Weller et al., 2018). This paucity of hibernacula necessitates identification and monitoring of alternate roosts, such as maternity colonies, for understanding long‐term population dynamics (Kunz & Reynolds, 2003). Maternity colonies provide high densities of bats for population monitoring, but neglect portions of the population, such as males, and seasonal roost and resource requirements (Weller et al., 2009). However, until western biologists are able to locate winter roosts with large numbers of bats, monitoring summer colonies may be an accessible, ecologically important alternative. Additionally, monitoring these roosts can produce seasonal (overwinter and oversummer) survival estimates at building roosts that are vital for reproduction and roosting habitat (Johnson et al., 2019; Voigt et al., 2016), and continued estimation of survival is critical for understanding the population dynamics of species susceptible to WNS (Frick, Reynolds, et al., 2010).

The high estimated rates of survival for long‐lived mammals such as *M. lucifugus* are not surprising, but most estimates of apparent survival are lower than true survival because they are confounded by some level of emigration (Boyles & Brack, 2009; Sandercock, 2006). This was the case for the *M. lucifugus* populations in this study. Through a separate radiotelemetry study, we found adult females using multiple‐day roosts during the maternity season (unpublished data). Additionally, we found six PIT‐tagged *M. lucifugus* from the house roost frequenting another maternity roost 3.2 km away. *Myotis lucifugus* are known to roost in networks of proximate summer roosts (Olson & Barclay, 2013), and efforts using telemetry and outreach to local landowners have uncovered additional roosts within several km of the house roost. Future attempts to install PIT‐tag‐reading antennas at these alternate roosts can clarify temporary emigration, and this additional information can be used to better estimate true survival and abundance, and refine estimates of detection probability (Dudgeon et al., 2015).

Several lines of evidence suggest the barn and the house roosts serve different requirements for *M. lucifugus*. We believe the house roost is a traditional maternity colony and the barn roost provides day‐ and night‐roosting habitat, in addition to maternity roost habitat. Because the barn has many openings, internal temperatures rarely matched the high temperatures documented at the house roost (high of 53°C) and at other maternity roosts (Webber & Willis, 2018). The barn provides protection from the elements and warm temperatures (high of 37°C) that match the conditions of other communal night roosts (Barclay, 1982). Additionally, the abundances at the house roost were relatively stable, while abundances at the barn roost showed substantial seasonal fluctuations. Female adult abundance peaked in early summer, and female juveniles increased in late summer. Anthony et al. (1981) documented late‐summer spikes in abundance at barn night roosts after young became volant. The barn may be used predominantly by pregnant females early in the summer, and then by postlactating, nonreproductive adults, and juveniles in late summer (Anthony et al., 1981). Further evidence the barn roost is used as a transient night roost comes from the lower estimates of site fidelity compared with the house roost. Lastly, recapture probability remains high at the house roost, while recapture probability decreases over time at the barn roost. Breeding females consistently return to the house roost throughout the summer, while early‐summer visitors at the barn may be pregnant individuals from nearby maternity colonies stopping over between feeding forays (Henry et al., 2002). Buildings are critical roosting resources for bats (Voigt et al., 2016) and may have greater significance for bats at higher elevations and latitudes where roosts with optimal temperatures may be less available (Johnson et al., 2019). Building roosts satisfy a host of roosting requirements for different ages, sexes, and species and should be conserved, especially as other roosts become scarcer or experience greater disturbance (Knight & Jones, 2009; Kunz & Reynolds, 2003; Voigt et al., 2016).

A multifaceted approach to monitoring *M. lucifugus* populations that includes a host of roost types will provide a better understanding of population dynamics, natural history, and habitat needs (Weller et al., 2009). Maternity colony monitoring can provide high sample sizes for estimating dynamics of reproductive female populations, but will neglect males, and because of the inability to predict when young bats become volant, juveniles can be undersampled, leading to poorly estimated parameters. Monitoring summer roosts that satisfy various roosting needs, such as the barn roost, allows access to volant young (307 versus 82 at the house roost; Table 1) and adult males, and provides better understanding of night‐roosting habitat needs. A strategy of monitoring different roosts throughout the year will provide a more‐complete understanding of regional *M. lucifugus* population dynamics.

A majority (83%) of PIT‐tagged bats were detected at antennas, which greatly exceeded the reencounter rate from manual recaptures, and provided consistent opportunities for detection throughout the year without the need for capturing and handling individuals. This noninvasive recapture method is valuable for detecting elusive species, improving estimates of population parameters, reducing stress to organisms, reducing the bias handling stress imparts on parameter estimates, and reducing the behavioral response bats can have to capture and that response's impact on detection probability (Ellison et al., 2007; Rigby et al., 2011). This technology is not without challenges, however, as PIT tags can be shed by individuals (pers. obs.), with some species being more susceptible to tag loss (Rigby et al., 2011). Although we only found three shed tags, it is likely bats have lost other tags. Interestingly, one tag was retained for one year prior to being shed, which suggests that PIT tags can be shed long after the insertion site has healed. Although the robust design model is robust to some violations of assumptions, such as tag loss, it is important to better estimate tag loss and its impacts on parameter estimates. Tag loss is not unique to this tagging methodology, as banding can present similar loss and possibly greater physical stress to bats (O'Shea et al., 2004), but should be addressed in any mark–recapture study. Additionally, powering antennas at remote study sites can be challenging, requiring access to electrical outlets, large batteries that need to be recharged periodically, or establishment of solar panel system. During several occasions, our power supplies were interrupted either by cattle that unplugged the unit, wildlife that tripped the connections between solar panels and batteries, or inadequate power from solar panel systems.

In addition to providing initial population parameters and an understanding of the populations at these roosts, survival and fidelity estimates can feed broader approaches that use multiple sources of demography data, such as integrated population models (Tempel et al., 2014). Much progress has been made to understand landscape‐scale population dynamics using hierarchical occupancy models, and these models are powerful tools for understanding population trends, and directing conservation effort (Rodhouse et al., 2019). However, local mark–recapture analyses may be more sensitive to population dynamics and are important for verifying projected or estimated landscape‐scale population changes (Conner et al., 2016). Continued effort to verify the relationship between occupancy and abundance is critical for making management decisions for North American bat populations (Tempel & Gutiérrez, 2013).

## CONFLICTS OF INTEREST

The authors have declared that no competing interests exist.

## AUTHORS' CONTRIBUTIONS

RAS, JLS: Conception of the ideas and design of methodology. RAS, JLS: Data; collection RAS: Data analysis; writing of the manuscript. RAS, JLS: Drafts; final approval for publication.

## Data Availability

The data that support the findings of this study are documented and archived in the Dryad Digital Repository (https://doi.org/10.5061/dryad.sj3tx964r).
